# Rab14 specifies the apical membrane through Arf6-mediated regulation of lipid domains and Cdc42

**DOI:** 10.1038/srep38249

**Published:** 2016-11-30

**Authors:** Ruifeng Lu, Jean M. Wilson

**Affiliations:** 1 Department of Cellular and Molecular Medicine, University of Arizona, Tucson, AZ 85724, USA.

## Abstract

The generation of cell polarity is essential for the development of multi-cellular organisms as well as for the function of epithelial organs in the mature animal. Small GTPases regulate the establishment and maintenance of polarity through effects on cytoskeleton, membrane trafficking, and signaling. Using short-term 3-dimensional culture of MDCK cells, we find that the small GTPase Rab14 is required for apical membrane specification. Rab14 knockdown results in disruption of polarized lipid domains and failure of the Par/aPKC/Cdc42 polarity complex to localize to the apical membrane. These effects are mediated through tight control of lipid localization, as overexpression of the phosphatidylinositol 4-phosphate 5-kinase α [PtdIns(4)P5K] activator Arf6 or PtdIns(4)P5K alone, or treatment with the phosphatidylinositol 3-kinase (PtdInsI3K) inhibitor wortmannin, rescued the multiple-apical domain phenotype observed after Rab14 knockdown. Rab14 also co-immunoprecipitates and colocalizes with the small GTPase Cdc42, and Rab14 knockdown results in increased Cdc42 activity. Furthermore, Rab14 regulates trafficking of vesicles to the apical domain, mitotic spindle orientation, and midbody position, consistent with Rab14’s reported localization to the midbody as well as its effects upon Cdc42. These results position Rab14 at the top of a molecular cascade that regulates the establishment of cell polarity.

A fundamental function of epithelia is to provide a barrier between the inside of an organism and the outside world. Epithelial cells are highly polarized, with the apical plasma membrane facing the lumen and basolateral membrane facing neighboring cells and the extracellular matrix. This polarity provides a mechanism for directed transport of ions and metabolites, and serves a first line defense against pathogens. In genetic systems such *C. elegans* and *Drosophila*, several protein complexes have been identified that promote the establishment and maintenance of polarity^1^,^2^,^3^,^4^,^5^,^6^,^7^,^8^,^9^,^10^,^11^,^12^, and work in mammalian cells has found that these underlying principles are maintained throughout Metazoa^13^,^14^,^15^,^16^,^17^,^18^,^19^,^20^,^21^,^22^. These polarity complexes include the Par/aPKC, Scribble/Discs Large, and Crumbs complexes^2^,^15^,^20^,^22^. However, prior to the assembly of these complexes at the apical or basolateral domain, the presumptive apical domain is established by a signaling cascade involving the extracellular matrix, Rho and Rab family GTPases, and the generation of distinct apical and basolateral lipid domains^13^,^23^,^24^,^25^,^26^,^27^,^28^. These processes promote the recruitment of the polarity complexes and directed vesicular trafficking that reinforce this identity and culminate in the formation of an apical lumen^20^,^29^,^30^,^31^,^32^,^33^,^34^,^35^,^36^.

The MDCK cell line has been used extensively to decipher the molecular mediators of polarity establishment in mammalian cells, and this system has provided great insight into these processes^31^,^32^,^37^,^38^. The conversion of a non-polarized cell to a polarized cell requires the asymmetric distribution of both proteins and lipids, and recent results using this cell system suggest that this asymmetry may be generated by the location of the cleavage furrow^39^,^40^, similar to bud site selection in yeast^3^,^4^,^41^,^42^. Several small GTPases have been localized to this domain, including Arf6, Rab8, Rab14, and Rab35^43^,^44^,^45^,^46^ and these molecules have all been implicated in epithelial polarity^29^,^30^,^32^,^35^,^40^,^47^,^48^,^49^,^50^. In this work, we examined the role of Rab14 in the earliest events in apical membrane specification. Previously, we found that Rab14 is involved in trafficking to the apical domain and that knockdown in 3D culture results in multiple lumens^48^,^51^. Furthermore, we found that Rab14 interacts with the polarity regulator aPKC^49^. Rab14 localizes to the cleavage furrow^45^, positioning it, along with Rab8, Rab11 and Rab35, in the correct region to impact the establishment of polarity. We show here that Rab14-regulated events play a crucial role in the earliest events in apical membrane initiation site (AMIS) specification as well as the later membrane trafficking events that promote the formation of an open lumen. We report that Rab14 is essential for the formation of PtdIns(4,5)P_2_-enriched lipid domains that promote the recruitment of downstream effectors of polarity, including Par3, aPKC and Cdc42. This appears to be mediated through Arf6 and PtdIns(4)P 5-Kinase, as overexpression of these proteins rescues the Rab14 KD phenotype. Furthermore, Rab14 interacts with Cdc42 and modulates its activity. These results position Rab14 at the top of a molecular cascade that regulates the initiation of cell polarity.

## Results

**Figure 1:**
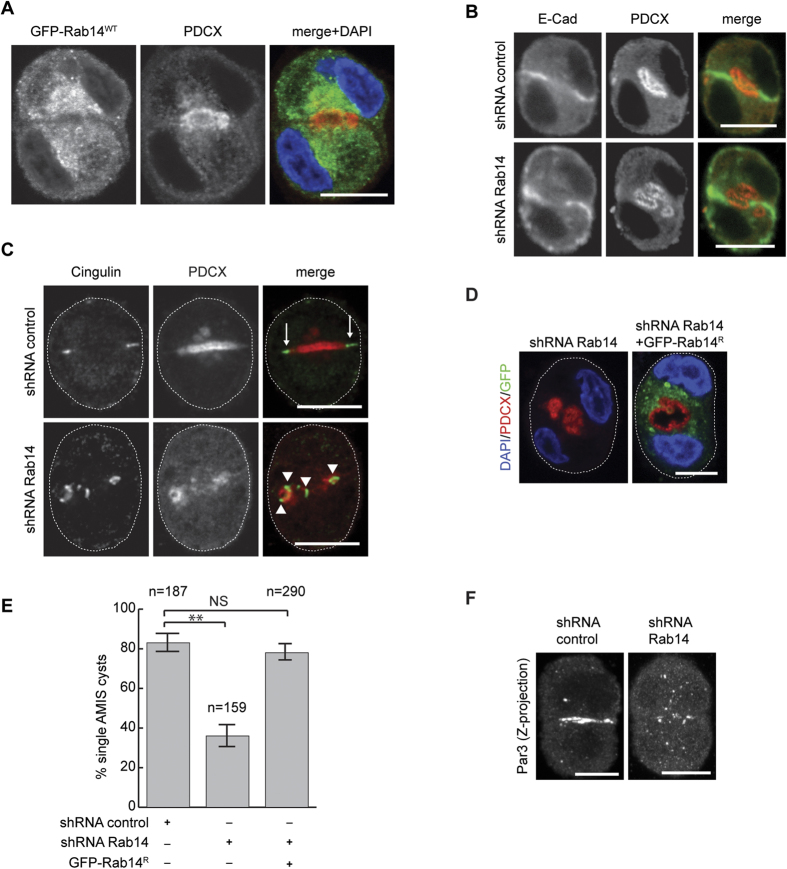
Rab14 is required for initiation of a single apical domain. (**A**) MDCK cells expressing GFP-Rab14 were plated in Matrigel and grown for 16 h, fixed and labeled for podocalyxin (PDCX, red). GFP-Rab14 is enriched in the cytoplasm between the nucleus and AMIS. DAPI labels the nucleus (blue). (**B**) shRNA mediated knock-down of Rab14 results in the development of several AMIS domains. Control cells have a single PDCX domain (red) that overlaps partially with E-cadherin (green). Rab14 knock down results in multiple distinct PDCX-positive domains. (**C**) The tight junction protein cingulin is mis-targeted after Rab14 knockdown. Cell pairs were labeled 16 h after plating with antibodies against PDCX (red) and cingulin (green). In control cells, cingulin localizes at the cell:cell interface (arrows). After Rab14 knockdown, cingulin is distributed at multiple sites at the interface (arrowheads). (**D**) Rescue of Rab14 knockdown. Rab14 KD cells were transfected with an shRNA-resistant Rab14-GFP. Expression of Rab14-GFP results in single lumen formation. (**E**) Quantification of single AMIS pairs after Rab14 KD and rescue. **p < 0.01, NS, not significant. (**F**) Par3 labeling after Rab14 KD. Par3 is localized to cell:cell contacts in control cells, but is distributed to cytoplasmic puncta after Rab14 KD. Scale bars, 10 μm.

Rab14 resides on endosomes and the TGN^48^,^52^,^53^,^54^,^55^. To define the localization of Rab14 in 3D cultures of cell pairs, we expressed GFP-Rab14 in MDCK cells. The “apical membrane initiation site” (AMIS) is located at the cell:cell interface at the two-cell stage^56^. At this stage, GFP-Rab14 localizes to tubule-vesicular structures between the nucleus and apical membrane (Fig. 1A). This localization is consistent with the finding that Rab14 traffics between the TGN and apical membrane and regulates apical targeting^48^ and also with the apical localization of Rab14 in two-dimensional culture^48^ as well as in mature cysts (Supplemental Fig. 1A).

### Rab14 is required for single AMIS specification

To determine if Rab14 is required for the earliest events in the specification of the apical membrane, we established stable Rab14 knockdown (Rab14 KD) MDCK cell lines using shRNA (Supplemental Fig. 1B,C^51^). The AMIS forms at the cell:cell contact, which then expands to form the “pre-apical patch” (PAP). Labeling of control cell pairs with antibodies against podocalyxin or cingulin 16 hours after plating demonstrates single AMIS or PAP sites (Fig. 1B,C). However, aberrant AMIS and multiple PAPs form in Rab14 KD cell pairs (Fig. 1B,C). This is not due to an off-target effect, as expression of shRNA resistant Rab14 rescued the AMIS formation defect (Fig. 1D,E). The polarity protein Par3 localizes to the AMIS early in the establishment of polarity^29^. Labeling cell pairs at this stage shows that Par3 is mislocalized to puncta in the cytoplasm after Rab14 knockdown (Fig. 1F). However, the protein level of Par3 is unchanged (Supplementary Figure 1D). These results suggest that Rab14 is required for the specification of a single AMIS during the early establishment of polarity in MDCK cells.

### Rab14 is required for the establishment of polarized lipid domains

**Figure 2:**
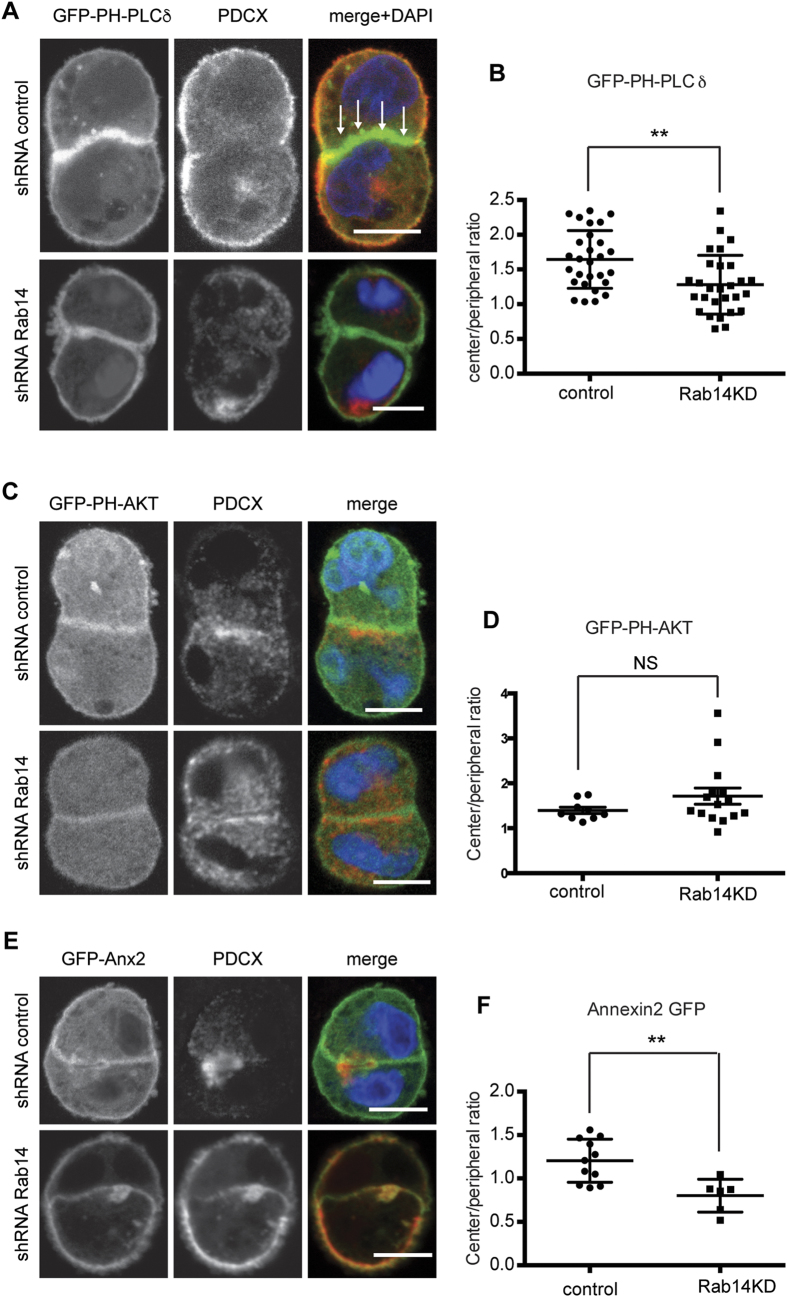
Rab14 is required for the establishment of PtdIns(4,5)P_2_-enriched lipid domains. (**A**) Cells were transfected with PLCδ-PH-GFP to probe for PtdIns(4,5)P_2_ and labeled for podocalyxin (red). In control cells, the signal is enriched at cell-cell contacts (arrows). Rab14 KD results in loss of this enrichment. (**B**) Quantification of **A.** Cell pairs were analyzed to determine the ratio of signal at the cell:cell interface compared to the signal at the peripheral membrane. Rab14 knockdown results in a significant loss of enrichment of probe at the cell:cell junction. (**C,D**) Cells transfected with AKT-PH-GFP to probe for PtdIns(3,4,5)P_3_ and labeled for podocalyxin. There are no differences in probe distribution between control and Rab14 KD cell pairs. (**E,F**) Cells were transfected with GFP-Anx2 and labeled for podocalyxin. There is decreased concentration of Anx2 at the cell:cell interface after Rab14 KD. **p < 0.002. NS, not significant. Scale bars, 10 μm. DAPI, nuclei (blue).

PTEN activity is thought to promote the enrichment of phosphoinositol (4,5) phosphate [PtdIns(4,5)P_2_] at the apical plasma membrane, resulting in recruitment of the Par6/aPKC polarity complex to the apical membrane^27^. We showed previously that knockdown of Rab14 results in mislocalization of aPKC to the basal domain in mature MDCK cell cysts^49^, and labeling of cell pairs with antibody against aPKC showed displacement of aPKC from the cell:cell interface after Rab14 KD (Supplemental Fig. 1E). To determine if Rab14 impacts events upstream of aPKC recruitment, we used probes of lipid domains to examine the localization of PtdIns(4,5)P_2_ in Rab14 KD cell pairs at these early stages of apical membrane specification. The pleckstrin homology (PH) domain of phospholipase D fused to GFP (PHD-GFP) was used as a marker for PtdIns(4,5)P_2_^57^. As shown in Fig. 2 and reported by others^27^, PHD-GFP is enriched at cell-cell contacts at the two-cell stage. In contrast, PHD-GFP is distributed evenly along cell-cell contacts and the peripheral membrane in Rab14 KD pairs (Fig. 2A). Quantification using plot profile analysis demonstrates a loss of enrichment of PtdIns(4,5)P_2_ at cell-cell contacts after Rab14 KD (Fig. 2B).

We next used PH-Akt-GFP to examine the localization of phosphatidylinositol 3,4,5 phosphate (PtdIns(3,4,5)P_3_) in control and Rab14 KD pairs^27^. We found that PH-Akt-GFP localized to both cell-cell contacts and the peripheral membrane, with a slightly higher concentration at cell-cell contacts in both wild type and Rab14KD cells (Fig. 2C). Consistent with this finding, quantification of the distribution of this probe does not show a significant difference between control and Rab14 KD pairs (Fig. 2D).

PtdIns(4,5)P_2_ recruits Annexin 2 (Anx2) to the plasma membrane^57^ and the binding of Anx2 to this phospholipid is important for apical membrane specification^27^. Furthermore, Anx2 binds to Rab14^58^. To determine if Rab14 KD impacts Anx2 recruitment to the AMIS, we expressed Anx2-GFP in control and Rab14 KD cell pairs. In control cells, there was an enrichment of Anx2 at cell-cell contacts (Fig. 2E). However, after Rab14 KD, Anx2 was no longer enriched in this region (Fig. 2E,F). Furthermore, labeling of endogenous of Anx2 showed decreased enrichment of this protein at cell-cell contacts after Rab14 KD (Supplemental Fig. 2).

### Expression of the small GTPase Arf6 rescues Rab14 knockdown

**Figure 3:**
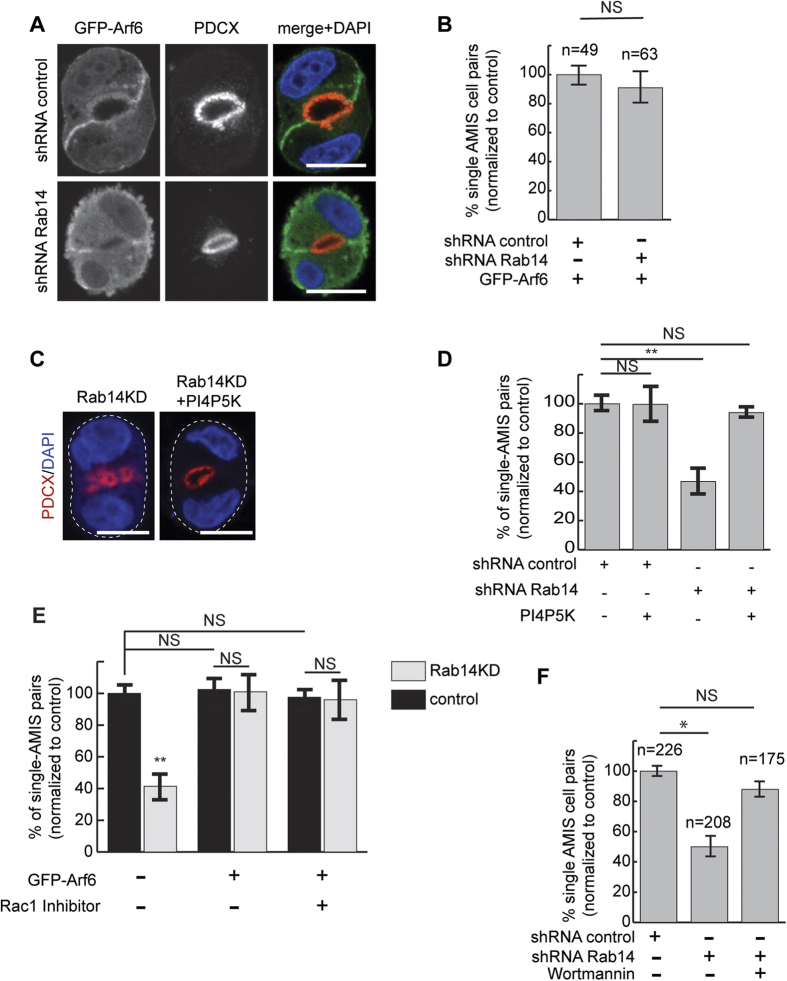
(**A,B**) Expression of GFP-Arf6 rescues the effects of Rab14 KD on single lumen formation. Control and Rab14 KD cells were transfected with GFP-Arf6 and labeled for podocalyxin (red). Expression of GFP-Arf6 results in normal single lumen formation in Rab14 KD cells. (**C,D**) Expression of PtdIns-4-P5 kinase rescues Rab14 knockdown. Control and Rab14 KD cells were transfected with PtdIns-4-P5 kinase and labeled for podocalyxin. Expression of PtdIns-4-P5 kinase after Rab14 KD results in single AMIS formation. **p < 0.001. (**E**) The effects of GFP-Arf6 on single lumen formation are not mediated through Rac1 activation. Incubation of Rab14 KD cells with an inhibitor of Rac1 has no effect upon Arf6-mediated rescue. **p < 0.001, NS, not significant. (**F**) Wortmannin treatment rescues the Rab14 KD multi-AMIS phenotype. *p < 0.05, NS, not significant. Scale bars for (**A,C**), 10 μm. DAPI, nuclei (blue).

These results place Rab14 at the beginning of a signaling cascade that begins with changes in phospholipid levels at the cell-cell interface. Changes in lipid levels may be regulated by the activity of either lipid kinases or lipid phosphatases, and both activities have been implicated in the establishment of polarity^13^,^25^,^27^,^28^,^59^,^60^. The small GTPase Arf6 activates PtdIns(4)P 5-kinase, resulting in increased PtdIns(4,5)P_2_^61^. In addition, Arf6 regulates apical membrane traffic and is localized to the cleavage furrow, positioning it as a potential regulator of apical specification. To determine if Arf6 could participate in this polarity pathway, we over-expressed GFP-Arf6 in Rab14 KD and control cells. Arf6-GFP localizes to the cell:cell junction in MDCK cell pairs (Supplemental Fig. 3A). Furthermore, expression of Arf6 rescues the multiple AMIS phenotype observed after Rab14 KD (Fig. 3A,B). Interestingly, in both control and Rab14 knockdown pairs expressing Arf6, the AMIS/PAP is substantially larger, consistent with increased PtdIns(4,5)P_2_ at this domain (compare with control KD Figs 1, 2 and 3).

Arf6 can activate both Rac1 and PtdIns(4)P 5-kinase. To test if Arf6 could be acting through stimulation of the lipid kinase, we over-expressed PtdIns(4)P 5-kinase in Rab14 KD and control cells. As shown in Fig. 3C,D, overexpression of PtdIns(4)P 5-kinase also rescues the multiple AMIS phenotype, suggesting that the effects on Arf6 after Rab14 KD are mediated through the lipid kinase.

To ask if the effects of Arf6 could also be mediated by activation of Rac1, we utilized a Rac1 inhibitor in Rab14 KD cells with and without overexpression of Arf6. As shown in Fig. 3, there is no change in the rescue of AMIS formation in Rab14 KD/Arf6 transfected pairs in the presence of the Rac1 inhibitor. Control experiments to confirm activity of the inhibitor demonstrate that this treatment nearly eliminated AMIS formation in wild type cell pairs (Supplemental Fig. 3). These results suggest that Arf6 is acting through the lipid kinase rather than Rac1 pathway. Furthermore, they suggest that the activity of Rac1 required for AMIS formation is upstream of the lipid kinase.

We next measured the levels of phosphorylated Akt, which can serve as an indicator of either increased PtdIns-3-kinase activity or decreased PTEN activity. P-Akt is slightly increased after Rab14 knockdown (Supplemental Fig. 3). To determine if the defect of AMIS specification after Rab14 KD could also result from increased PtdIns-3-kinase activity, we treated cell pairs with wortmannin at plating and quantified AMIS formation. This treatment restored nearly normal AMIS formation in Rab14KD cells (Fig. 3F). Together, these results suggest that Rab14 modulates the effectors of lipid kinases and thus the establishment of these lipid domains early in AMIS specification.

### Rab14 interacts with Cdc42

**Figure 4:**
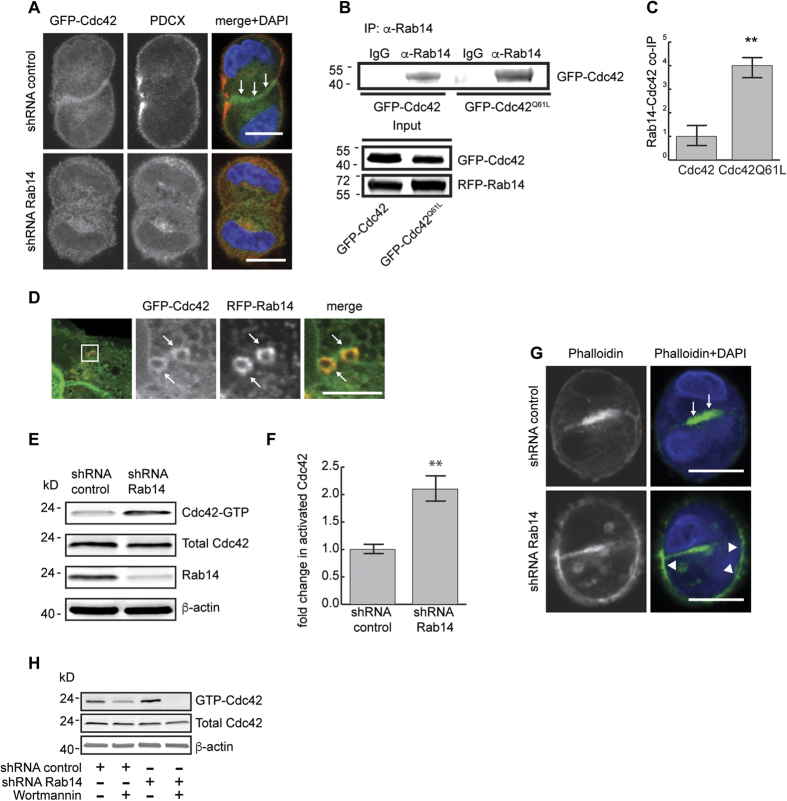
Rab14 modulates Cdc42 activity and localization. (**A**) Cells transfected with GFP-Cdc42 and labeled for podocalyxin. In control cells, GFP-Cdc42 is enriched at cell contacts (arrows). Rab14 KD results in loss of enrichment at this domain. (**B**) Co-immunoprecipitation of RFP-Rab14 and GFP-Cdc42 or RFP-Rab14 and GFP-Cdc42Q61L. GFP-Cdc42 WT and GFP-Cdc42 Q61L co-immunoprecipitate with Rab14 and there is increased co-immunoprecipitation of GFP-Cdc42Q61L. (**C**) Quantification of 3 independent co-immunoprecipitation experiments. **p < 0.001. (**D**) Cells co-transfected with RFP-Rab14 and GFP-Cdc42. RFP-Rab14 and GFP-Cdc42 colocalize on some endosomes at the periphery of the cell (arrows). (**E,F**) Analysis of Cdc42-GTP levels after Rab14 KD. Rab14 KD results in more Cdc42-GTP without a change in total Cdc42. **p < 0.001. (**G**) Control and Rab14 knockdown cell pairs were labeled with rhodamine-phalloidin. Image was pseudocolored green. Control cells have enriched phalloidin labeling at the AMIS (arrows) whereas Rab14 KD results in increased filamentous actin at the cell periphery (arrowheads). (**H**) Wortmannin treatment reverses the activation of Cdc42. Control and Rab14 KD cells were incubated with wortmannin and active Cdc42 was pulled down with PBD-beads. There is decreased active Cdc42 after wortmannin treatment. Scale bars, (**A**,**G**), 10 μm, (**D**), 5 μm. DAPI, nuclei (blue).

Annexin2 targets activated Cdc42 to the AMIS^27^. Cdc42 then recruits additional factors that culminate in epithelial cyst morphogenesis^27^. Since Anx2 is mislocalized with Rab14 knockdown, we next used Cdc42-GFP to ask if the localization of Cdc42 was also impacted. Eight hours after plating, Cdc42-GFP was enriched at cell-cell contacts in control cell pairs. However, Rab14 KD resulted in the loss of the enrichment of Cdc42 at sites of cell-cell contact (Fig. 4A).

We showed previously that Rab14 interacts directly with aPKC^49^. Cdc42 is part of the polarity complex that includes aPKC^5^,^17^. To determine if Rab14 could also form a complex with Cdc42, we over-expressed RFP-Rab14 and Cdc42-GFP followed by immunoprecipitation and Western blotting. As shown in Fig. 4B, Rab14 co-immunoprecipitates with Cdc42, and there was increased co-immunoprecipitation of Rab14 with the constitutively active form, Cdc42-Q61L (Fig. 4C). Furthermore, we imaged RFP-Rab14 and Cdc42-GFP and find that they colocalize on intracellular vesicles at the cell periphery (Fig. 4D).

Knock down of Rab8a results in decreased levels of active Cdc42^29^,^62^. The increased binding of Cdc42-Q61L to Rab14 suggests that Rab14 may also regulate Cdc42 activity. To test this, we used the p21-binding domain of PAK1 fused to GST to pull-down active Cdc42 in control and Rab14 knockdown cells. Although the total protein levels of Cdc42 were not changed by Rab14 knockdown (Fig. 4E), the amount of active Cdc42 increased in Rab14 KD cells (Fig. 4F). Furthermore, staining of cell pairs with phalloidin shows filamentous actin at both the cell-cell junction and the periphery after Rab14 KD, contrasting with control cells where phalloidin staining is restricted to the AMIS (Fig. 4G). These results suggest that the increased activity and mis-localization of Cdc42 by Rab14 knockdown results in aberrant Cdc42 activity at the cell periphery.

Cdc42 can be activated by increased PtdIns-3-kinase^63^,^64^,^65^,^66^. To test if the increased activation of Cdc42 in Rab14 KD cells could be mediated by increased PtdIns-3-kinase activity, we treated Rab14 KD cells with wortmannin, followed by PBD pull-down of active Cdc42. As expected, wortmannin treatment decreased the activation of Cdc42 in control cells (Fig. 4H). In addition, wortmannin also decreased Cdc42 activation in Rab14 KD cells, suggesting that the observed increase in Cdc42-GTP could be due to changes in PtdIns 3,4,5P_3_ levels.

### Rab14 knockdown affects trafficking to the apical domain

Rab14 has important roles in trafficking between the TGN, endosomes, and the plasma membrane^48^,^51^,^52^,^53^,^55^,^67^,^68^. Podocalyxin traffics from the periphery of cells to the apical domain and this trafficking is required for normal lumen formation^31^. To assess the role of Rab14 in this process, we incubated cell pairs with anti-podocalyxin at 37 °C for 20 minutes. As shown in Supplemental Fig. 4, we observe 3 main phenotypes: peripheral podocalyxin, a single PAP, or multiple AMIS/PAPs. Quantification shows that Rab14 KD results in increased peripheral labeling as well as multiple PAP profiles compared to control pairs. To determine if Rab14 KD causes a general decrease in endocytosis, we performed internalization assays with fluorescent dextran. In fact, Rab14 KD results in increased uptake of soluble tracer (Supplemental Fig. 4), indicating that the decreased transcytosis of podocalyxin after Rab14KD is not due to a global defect in endocytosis.

**Figure 5:**
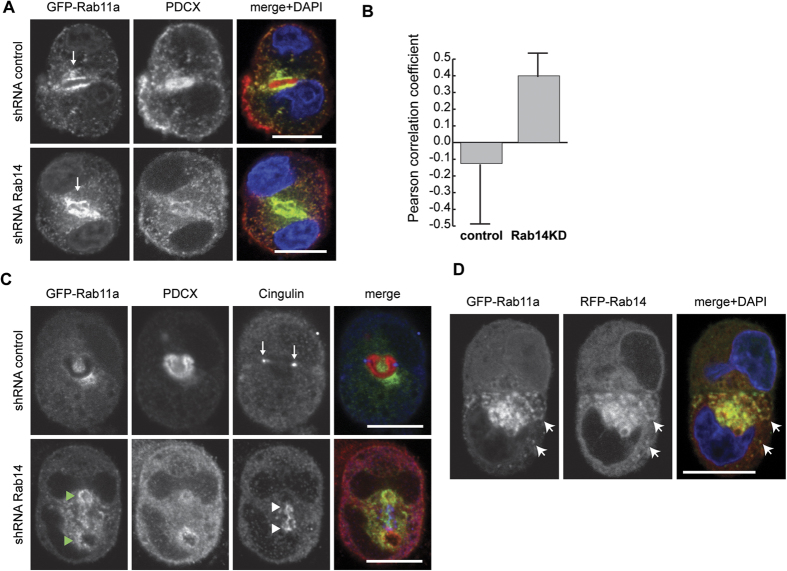
(**A**) Rab14 KD does not impact the distribution of GFP-Rab11a. Cells over-expressing GFP-Rab11 were labeled for podocalyxin (red). GFP-Rab11a surrounds the apical lumen in both control and Rab14 KD pairs (arrows) and is present in cytoplasmic puncta. However, Rab14 KD results increased cytoplasmic podocalyxin (middle panel). (**B**) Pearson’s correlation coefficient analysis of peripheral GFP-Rab11a and podocalyxin colocalization. No correlation was observed in control cells. However, Rab14 KD results in an increase in the Pearson’s correlation of endosomal podocalyxin and GFP-Rab11a. (**C**) Cell pairs over-expressing GFP-Rab11a were co-labeled with cingulin (blue) and podocalyxin (red). In control pairs, cingulin localized to tight junctions (arrows) while GFP-Rab11a surrounds the AMIS. In Rab14 KD pairs, GFP-Rab11 (green arrowheads) accumulates near cingulin (white arrowheads) but there is decreased AMIS-associated podocalyxin. (**D**) Cells co-transfected with GFP-Rab11a and RFP-Rab14. GFP-Rab11a and RFP-Rab14 colocalize near the AMIS and in the periphery (arrows). Scale bars, 10 μm. DAPI, nuclei (blue).

The small GTPase Rab11a is a key component of the apical recycling endosome that aids in the trafficking of proteins to the midbody as well as the luminal surface of polarized epithelial cells^29^,^35^,^69^,^70^,^71^. To determine if Rab14 KD impacts the trafficking of Rab11a, we examined the localization of GFP-Rab11a in cell pairs. Under control conditions, GFP-Rab11a localizes to the PAP (Fig. 5A) along with podocalyxin. GFP-Rab11a also localized to the PAP in Rab14KD cells, but a majority of podocalyxin remained at the periphery or in cytoplasmic puncta (Fig. 5A). This suggests that Rab14 is required for transport of podocalyxin from Rab11a endosomes to the apical plasma membrane. To determine if Rab14 knockdown results in delayed endosomal trafficking from the periphery to the PAP, we quantified the colocalization of Rab11a and podocalyxin near the basal membrane. This analysis shows that podocalyxin in the peripheral cytoplasm colocalized with GFP-Rab11a in Rab14 KD cells but not in control cells (Fig. 5B), supporting the idea that Rab14 is required to traffic these vesicles to the apical domain. Alternatively, Cdc42 activity is required for trafficking of podocalyxin from intracellular vesicles to the AMIS^29^. It may be that the effects of Rab14 KD on Cdc42 are limiting trafficking from these vesicles.

To further test the impact of Rab14 KD on GFP-Rab11a distribution, we labeled cell pairs with the tight junction protein Cingulin. GFP-Rab11a accumulates around multiple cingulin-positive AMIS in Rab14 KD pairs (Fig. 5C), again indicating that Rab11a targeting to the forming AMIS is independent of Rab14.

Rab14 has been proposed to regulate the fast recycling pathway while Rab11a regulates a slower recycling pathway^53^. To examine if Rab14 and Rab11a could cooperate on a single population endosomes, we over-expressed GFP-Rab11a and RFP-Rab14 in cell pairs. Rab14 and Rab11 colocalize on some endosomes close to the apical domain as well as in the periphery (Fig. 5D). Therefore, while these Rabs may regulate distinct parts of the pathway, it may be from a common endosome.

### Rab14 knockdown results in defects in the mitotic spindle and midbody

**Figure 6:**
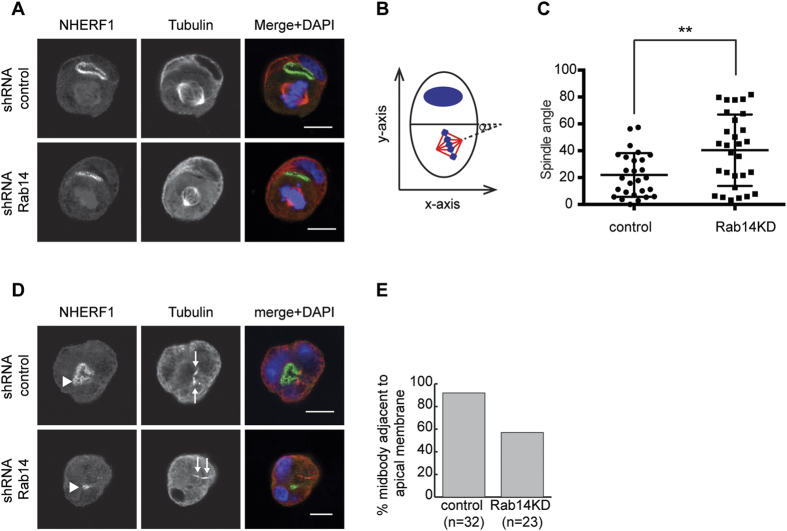
Rab14 KD results in random spindle angle and midbody position during mitosis. (**A**) Cells pairs were labeled with antibodies against NHERF1 and tubulin and spindle angle of cell pairs was analyzed. (**B**) Diagram depicting how spindle angle was measured. (**C**) Quantification of spindle angles. In control pairs, the average spindle angle is 22°. Rab14 KD results in a random distribution with an average near 45°. (**D**) Cell pairs were labeled with NHERF1 (green) and tubulin (red). The position of the midbody (arrows) relative to the apical membrane labeled by NHERF1 (arrowheads) was analyzed. (**E**) Quantification shows the percentage of pairs with the midbody next to apical the membrane. Rab14 KD results in misplacement of the midbody. Scale bars, 10 μm.

Aberrant mitotic spindle orientation or deposition of the midbody results in the formation of ectopic lumens^16^,^34^ and the spatiotemporal regulation Cdc42 is required for both of these processes^16^,^72^,^73^,^74^,^75^,^76^. Since Cdc42 is hyperactive and mislocalized in Rab14 KD cells, we tested whether mitotic spindle orientation was changed after Rab14 KD. We labeled the mitotic spindles using anti-tubulin antibodies and measured the spindle angle (Fig. 6A). In control pairs, the mitotic spindles orient parallel to cell-cell contacts (median angle 21°, Fig. 6B,C). However, Rab14 KD results in random spindle orientation. Further, midbody positioning is essential for single lumen formation and is also regulated by Cdc42^16^,^38^,^77^,^78^. To determine if Rab14KD impacts midbody positioning, we quantified the position of the midbody in control and Rab14 KD pairs. In control pairs, the midbody is positioned next to apical domain in more than 90% of the cell pairs (n = 32), while Rab14KD results in midbody positioning next to one of multiple apical domains in 58% of pairs (n = 23). This result likely underestimates the displacement of the midbody, since these pairs have multiple forming apical domains.

## Discussion

Here we report that the small GTPase Rab14 is required for the early events in AMIS specification, and that this may be due to modulation of lipid domains that precedes the recruitment of polarity proteins. Expression of Arf6 or PtdIns4P5 kinase or incubation with a PtdIns-3-kinase inhibitor rescues Rab14 knockdown, suggesting that Rab14 plays an essential role in maintaining the phospholipid content of the forming apical membrane. This phospholipid balance then promotes the recruitment of Cdc42 and provides the polarization cues that result in single lumen formation. We also find that Rab14 knockdown delays the delivery of podocalyxin to the developing AMIS, indicating that it functions in both early and late events in the establishment of polarity.

Recent results have demonstrated a role for the midbody as an initiating site for polarity specification, and both Rab11a and Rab35 are known to be involved in trafficking to this domain^40^,^77^,^79^. This trafficking and AMIS specification are intimately linked. Previous work has found that apical specification is mediated through the enrichment of specific phosphoinositides together with assembly of the polarity complexes at the apical domain^27^. This is followed by polarized membrane trafficking to enlarge and reinforce the apical domain. We find that Rab14 KD results in loss of apical enrichment of PtdIns(4,5)P_2_. PtdIns(4,5)P_2_ is a key determinant of the apical membrane domain, and disruption of this localization by delivery of exogenous PtdIns(4,5)P_2_ to cysts in culture results in inversion of polarized cysts^27^. PtdIns(4,5)P_2_ enrichment can be mediated by synthesis by PtdIns4P5 kinase or by dephosphorylation of PtdIns(3,4,5)P_3_ by PTEN. While PTEN has been shown to localize to the apical domain^59^, we find that over-expression of Arf6, which activates PtdIns4P5 kinase, or the PtdIns4P5 kinase alone, rescues AMIS specification in Rab14 KD cell pairs, suggesting that this pathway is an important component in the establishment of polarity. Interestingly, in neurons, Rab35 inactivates Arf6 through recruitment of its GAP to regulate neurite outgrowth^80^. It may be that Rab14 and Rab35 act in opposition to control phospholipid levels at the forming apical membrane.

We also find increased phosphorylation of Akt, indicating increased levels of PtdIns(3,4,5)P_3_ after Rab14 KD. This could be due to increased phosphorylation of PtdIns(4,5)P_2_ by PtdIns-3-kinase or decreased PTEN activity on PtdIns(3,4,5)P_3_. Our finding that treatment with wortmannin, a PtdIns-3-kinase inhibitor, rescues the Rab14 KD phenotype, suggests that Rab14 KD is not acting through PTEN. Furthermore, Par-3, which binds to PTEN and is recruited to the apical domain^27^, is also displaced after Rab14 KD, further supporting a model in which Rab14 initiates the pathway of polarity establishment independently of PTEN.

Cdc42 is a part of the polarity complex that is required for normal lumen formation. Manipulation of Cdc42 or its GEFs, results in cysts with multiple lumens^16^,^27^,^29^,^74^,^75^. The increased activation of Cdc42 observed after Rab14 KD could be due to several factors. We find that Rab14 and Cdc42 co-immunoprecipitate and co-localize in cells. Rab14 could recruit a Cdc42 GAP that promotes cycling of Cdc42 to regulate polarity. Alternatively, PtdIns-3-kinase has been implicated in Cdc42 activation, and changes in PtdIns(3,4,5)P3 levels could directly impact the activation of Cdc42^63^. It is important to note that knockdown of Rab8, another regulator of AMIS specification, results in decreased Cdc42 activity^29^,^62^. It is possible that Rab14 and Rab8 act antagonistically for the spatiotemporal regulation of Cdc42 during polarity establishment.

After establishment of the AMIS, the forming lumen expands through the directed targeting of vesicles to this domain, forming a “pre-apical patch” or PAP. This vesicular trafficking is mediated by Rabs 11a and 8a^29^, and recent results suggest that several Rabs participate in this trafficking^32^,^40^. We find that knockdown of Rab14 also impacts podocalyxin trafficking, resulting in delayed delivery to the PAP. Our results contrast with a recent report that suggests no effect of Rab14 KD on single lumen formation, although in that report the impact of Rab14 knockdown on single lumen specification was statistically significant^32^. The reason for our more substantial phenotype is unclear. However, the method for knockdown is different, as we used stable shRNA-mediated knockdown compared to siRNA. We are confident that our results are not due to an off-target effect, as expression of an shRNA-resistant form of Rab14 rescued the multiple lumen phenotype.

Current models of polarized trafficking involve Rab11a recruitment of Rab8a to endosomes, followed by binding of Cdc42 and targeting, via myosin Vb, to the apical domain^29^,^34^,^35^. Our results suggest that Rab14 is necessary for trafficking of podocalyxin to the apical endosomes, although these defects could reflect Rab14 effects upon Cdc42^29^. Rab11a and Rab14 are present in the same endosomal compartments at these early times in lumen formation. However, we do not observe colocalization of Rab14 and Rab8, suggesting that these domains are distinct during these early events (data not shown).

Cdc42 is a central regulator of mitosis and spindle orientation, and its spatial-temporal regulation is important for these functions^76^,^81^,^82^. Knockdown of Cdc42 in multicellular Caco2 cysts disrupts spindle orientation and midbody position, and both spindle orientation defects and midbody positioning contribute to the formation of ectopic lumens^16^. Here, we show that Rab14 KD results in displacement of Cdc42 from the cell-cell interface, as well as increased activation and displacement of the midbody. This may be due to Rab14 mediated changes in lipid composition or due to the effects on Cdc42. Regardless, these results position Rab14 as a central regulator of some of the earliest events in cell polarization.

Like Rabs 8a and 11a, which have been shown to be involved in the specification of the apical domain, Rab14 has been suggested to be an ancestral Rab. It is found in multiple eukaryotic taxa, and may comprise part of the Rab repertoire of the Last Eukaryotic Common Ancestor^83^,^84^. Our finding that Rab14 is responsible for early events in AMIS specification is consistent with an ancient role for this Rab.

## Methods

### Reagents

Cell culture reagents were purchased from Mediatech (Manassas, VA). General chemicals were purchased from Sigma-Aldrich (St. Louis, MO). The following primary antibodies were used: mouse anti-podocalyxin was a gift from Dr. Karl Matlin (University of Chicago); rabbit anti-PKCζ (Santa Cruz Biotechnology, Dallas, TX); mouse anti-tubulin, mouse anti-β-actin, rhodamine-phalloidin, rat anti-E-cadherin (Sigma), chicken anti-GFP (Life Technologies, Grand Island, NY); rabbit anti-NHERF1 (Abcam, Cambridge, MA); rabbit anti-Par3 (EMD Millipore, Billerica, MA); rabbit anti-cingulin was a gift from Dr. Rytis Prekeris (University of Colorado, Denver). Secondary antibodies were purchased from Molecular Probes/Thermo-Fisher (Eugene, OR). GFP-Cdc42 and GFP-Cdc42Q61L were purchased from Addgene (Cambridge, MA). GFP-PH-PLCδ, GFP-PH-Akt, and Annexin2-GFP plasmids were gifts from Dr. Keith Mostov (University of California, San Francisco). Arf6-GFP and PtdIns(4)P 5 K plasmids were described previously^85^,^86^. GFP-Rab11a, GFP-Rab14, and GFP-Rab14S25N were prepared as described^48^. Matrigel was purchased from Corning Life Sciences (Corning, NY).

### Cell culture

Cells were cultured in high-glucose DMEM supplemented with 10% fetal bovine serum, 1% nonessential amino acids at 37 °C, 5% CO_2_. Transfections were performed with Lipofectamine 2000 (Life Technologies, Carlsbad, CA) following the manufacturer protocol. Transfected cells were selected in DMEM containing 400 μg/mL G418 (Mediatech) for one week to establish stable lines. Transduction was performed as described using the same Rab14 shRNA constructs^51^. Knockdown was confirmed by immunoblotting and reconfirmed in each experimental series. For analysis of cell pairs, glass-bottom eight-well chambers were pre-coated with Matrigel (20 μL/chamber) on ice and then incubated at room temperature for 15 minutes. Single cell suspensions in 2% Matrigel were plated into each chamber. Cells were grown for 16 h, fixed, and labeled as described.

### Immunoblotting

Cells were lysed in RIPA buffer (20 mM Tris,100 mM NaCl, 1% Triton, 1% Na-deoxycholate, 0.1% SDS and EDTA-free protease inhibitors; Roche). After immunoblotting, membranes were scanned and quantified on an LI-COR Odyssey, (Lincoln, NE) using LI-COR 3.0 analytical software. β-actin was used as a loading control. Protein bands were normalized to in-lane β-actin. Normalized numbers were analyzed and plotted in Microsoft Excel. Statistics were from at least 3 loading replicates and each experiment was repeated at least 2 times.

### Immunofluorescence Microscopy

Cells were fixed in 2% paraformaldehyde at 37 °C for 30 minutes, washed and quenched by incubation in 50 mM NH_4_Cl/PBS. Cells were permeabilized and blocked with 0.5% saponin and 10% FBS in 1XPBS followed by incubation with primary antibody in 1XPBS, 0.5% saponin, 10%FBS overnight at 4 °C. After washing, cells were incubated with secondary antibodies for 8 hours at 4 °C. After washing, 40 μL Pro-Long Gold (Life Technologies) was added to each chamber. Images were acquired with an Olympus FluoView 1200 confocal microscope (Olympus, Tokyo, Japan) with a 60 × NA 1.43 oil immersion objective and identical imaging parameters were used within a single experiment. Images were processed and analyzed using Adobe Photoshop (Adobe, San Jose, CA) and ImageJ^87^,^88^,^89^.

### Uptake assays

For anti-podocalyxin uptake, cells were cultured for 16 hours and gp135 antibody (1:15) was added to the culture media. After 20 minutes at 37 °C, cells were fixed and labeled. For FITC-Dextran uptake, cells were cultured to 75% confluence in a 10-cm dish and media was replaced with DMEM containing 1 mg/mL 4KD FITC-Dextran (Sigma). After 20 minutes at 37 °C, cells were washed with 1XPBS, lysed and lysates were collected and centrifuged. 4 replicates were collected and fluorescein signal was read on a Varioskan Flash plate reader (Thermo Fisher Scientific, Waltham, MA).

### Co-immunoprecipitation

HEK cells were co-transfected with RFP-Rab14 and GFP-Cdc42 or GFP-Cdc42Q61L. 24 h after transfection, cells were lysed in Pierce Lysis Buffer (Thermo Scientific, Rockford, IL). Rabbit anti-Rab14 antibody was bound to Dynabeads (Life Technologies) and rabbit IgG was used as the control. After binding, the bead-antibody complex was mixed with cell lysates and incubated on a rotator at 4 °C for 1 hour. Beads were washed and eluted with SDS-PAGE sample buffer and immunoblotted with anti-GFP antibody.

### Cdc42 activation assay

Cells were lysed and 500 μg of lysate was mixed with PAK-PBD beads. PAK-PBD beads were pelleted by centrifugation, washed two times, eluted with SDS-PAGE sample buffer and analyzed by immunoblotting.

### Drug treatments

For treatment with wortmannin to assess Cdc42 activation, cells were treated for 3 h at 37 °C, followed by stimulation with insulin for the indicated times. Cells were lysed and analyzed for Cdc42 activation as described above. For analysis of cell pairs, cells were plated in Matrigel and then treated for 16 h in 200 nM wortmannin followed by fixation and labeling as indicated. For Rac1 inhibitor studies, cells were plated in 2% Matrigel containing 50 μm CAS 1177865–17–6 (EMD Millipore) and incubated for 16 h followed by fixation and labeling.

### Spindle angle measurement

Cysts were fixed and labeled with anti-tubulin and anti-NHERF1. Cysts at metaphase were imaged and used for spindle angle measurements as shown in Fig. 6B.

### Statistics

Each experiment was repeated independently at least twice with at least 3 replicates. Quantification data are presented as mean ± SEM. Statistical significance was calculated using a two-tailed unpaired student t-test (Microsoft Excel, Redmond, WA) at a 95% confidence interval.

## Additional Information

**How to cite this article**: Lu, R. and Wilson, J. M. Rab14 specifies the apical membrane through Arf6-mediated regulation of lipid domains and Cdc42. *Sci. Rep.* **6**, 38249; doi: 10.1038/srep38249 (2016).

**Publisher’s note:** Springer Nature remains neutral with regard to jurisdictional claims in published maps and institutional affiliations.

## Supplementary Material

Supplementary Information
