# Supplementary material for: Rab14 specifies the apical membrane through Arf6-mediated regulation of lipid domains and Cdc42
Source: Sci Rep. 2016 Nov 30;6:38249. doi: 10.1038/srep38249 (PMC5128791; doi:10.1038/srep38249)
Supplement: Supplementary Information [file srep38249-s1.pdf]

Supplemental Figure 1

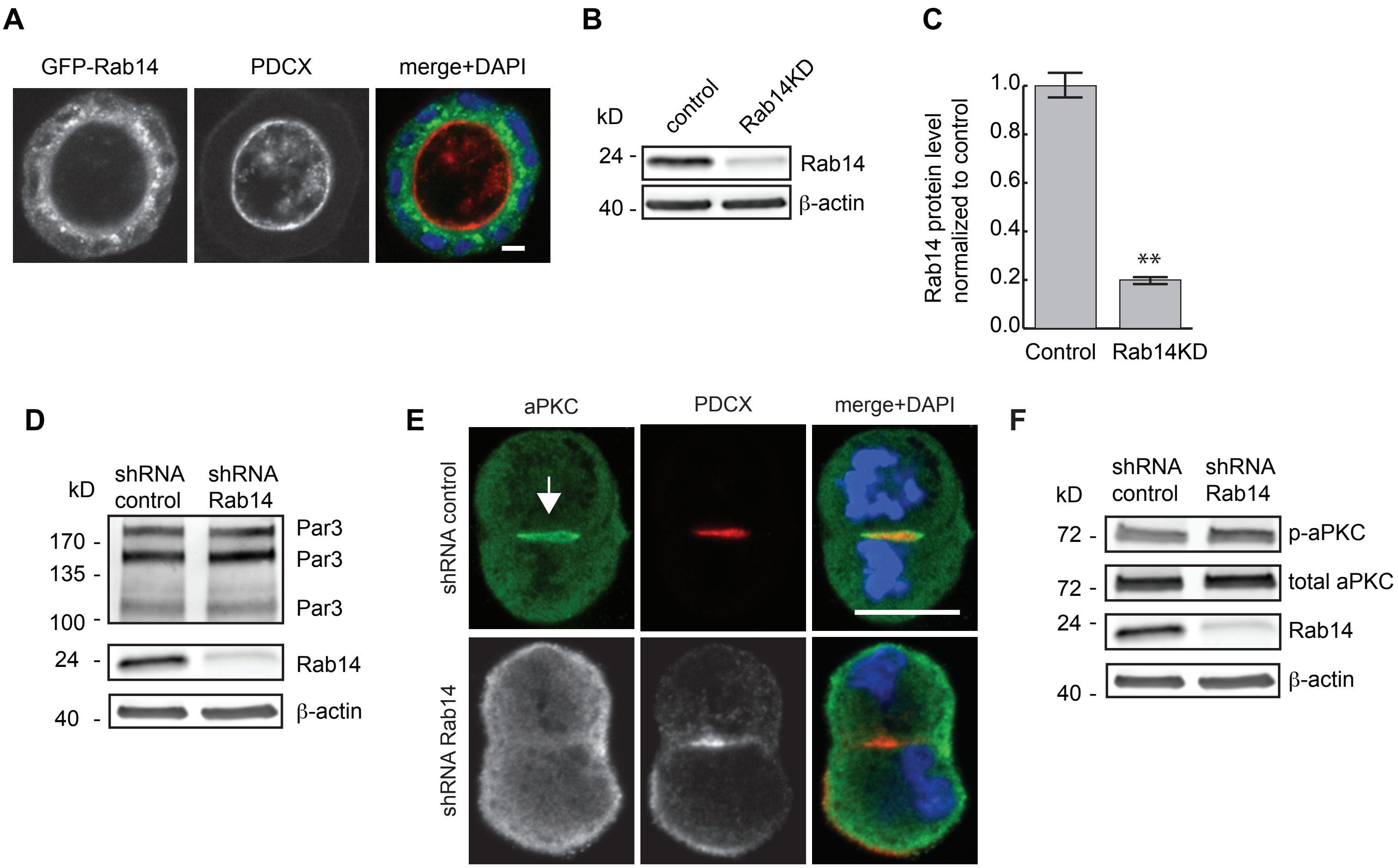

Supplemental Figure 2

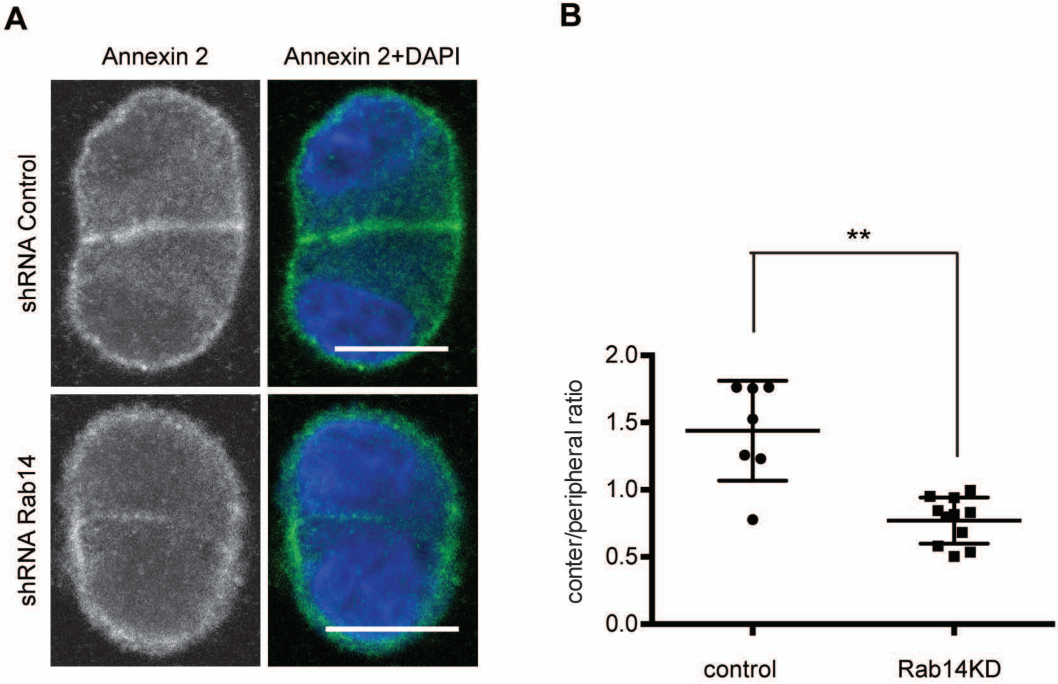

Supplemental Figure 3

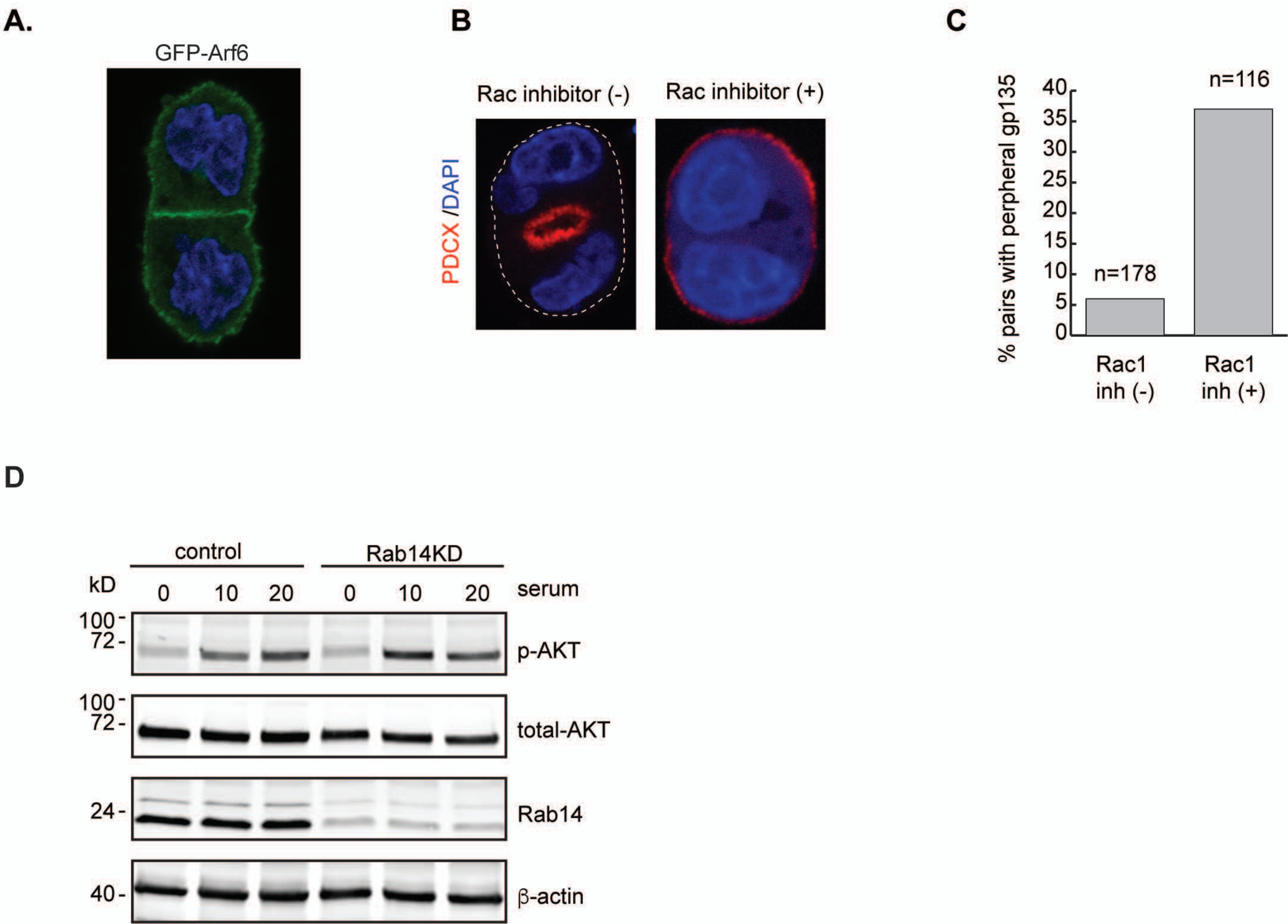

Supplemental Figure 4

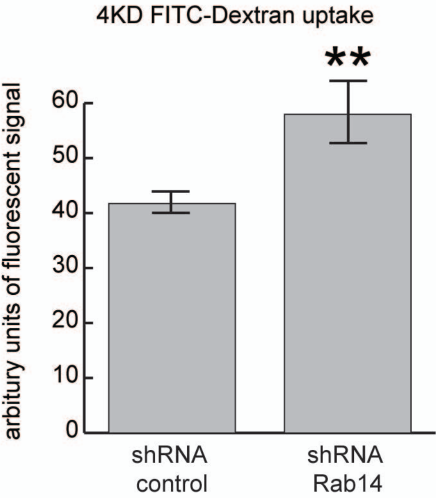

Rab14 specifies the apical membrane through Arf6-mediated regulation of lipid domains and Cdc42
Lu and Wilson

Supplemental Figure 1. A. Rab14 localizes to apical vesicles in mature cysts. Cells were transfected with GFP-Rab14 and fixed and labeled for podocalyxin (red) 4 days after plating. GFP-Rab14 localizes to tubulovesicular membranes adjacent to apical membrane. **B., C**. Rab14 protein level is reduced by Rab14 specific shRNA. Stable cell lines were infected with shRNA lentivirus against Rab14. Rab14 expression is decreased by more than 80%. **D**. Par-3 protein levels do not change after Rab14 KD. Rab14 KD results in displacement of aPKC from the forming AMIS. **F.** There is no change in aPKC levels after Rab14 KD. Scale bars, 10 µm. DAPI, nuclei (blue).

Supplemental Figure 2. A. Rab14 KD results in mis-localization of endogenous Anx2. **B.** Quantification of Anx2 distribution after Rab14 KD. Scale bars, 10 µm. **, $p<0.002$.

Supplemental Figure 3. A. Arf6-GFP localizes to cell:cell contacts. **B.** Incubation with Rac1 inhibitor inhibits transport of podocalyxin from the periphery to the AMIS. **C.** Quantification of effects of the Rac1 inhibitor on podocalyxin trafficking. **D.** Rab14 KD increases AKT phosphorylation. Cells were serum starved overnight and 20% serum was added. Cells were lysed 0, 10 or 20 minutes after the replacement of serum. Phospho-AKT was analyzed by Western Blot. There is increased P-AKT in Rab14 KD cells 10 minutes after stimulation. The figure is representative of 3 independent experiments.

Supplemental Figure 4. Rab14 KD increases fluid phase endocytosis. Cells were

incubated with 4KD FITC-dextran for 20 min at 37°C followed by lysis and quantification by fluorimetry. **, $p<0.001$.
